# Knowledge and attitudes towards contraceptives among adolescents and young adults

**DOI:** 10.1186/s40834-020-00144-3

**Published:** 2021-01-05

**Authors:** Aanchal Sharma, Edward McCabe, Sona Jani, Anthony Gonzalez, Seleshi Demissie, April Lee

**Affiliations:** 1 Department of Pediatrics, Staten Island University Hospital, Staten Island, NY USA; 2 Department of Developmental Medicine, Boston Childrens Hospital, Boston, MA USA; 3 Division of Adolescent Medicine, Staten Island University Hospital, Staten Island, NY USA; 4 Department of Research, Staten Island University Hospital, Staten Island, NY USA; 5 Biostatistics Unit, Feinstein Institute for Medical Research, Staten Island University Hospital, Staten Island, NY USA

**Keywords:** Contraception, Reproduction, IUD, Sexual health, Adolescent health

## Abstract

**Background:**

Despite endorsements supporting the use of intrauterine devices (IUDs) for adolescents and young adult women (AYA), they have limited knowledge about them Male partners can influence contraceptive decisions, however their perceived knowledge about IUDs is lower than their objective knowledge. We aim to establish current AYA baseline contraceptive knowledge and attitudes so providers can better target their sexual health educational interventions.

**Methods:**

Females and males, aged 13 to 23 years old, from our suburban adolescent clinic, completed an anonymous survey that assessed their knowledge and attitudes towards methods of contraception, with an emphasis on the IUD.

**Results:**

Completed surveys totaled 130 (99 females/31 males). Demographic results revealed 31.3% Black/African-American, 30.5% Latino/Hispanic, 17.6% White, 3.0% Asian, and 14.5% Other. The majority of participants (80%) were sexually active. The majority (69.5%) stated they/their partner were currently using a contraceptive method; only 2.6% used IUDs. Half of females (56.6%) and 10.1% of males had heard of IUDs. Despite this, male and female participants lacked knowledge regarding specific IUD facts. Of the participants who had used emergency contraception (EC), only 6.4% knew the copper IUD could be used for EC.

**Conclusion:**

Contraceptive knowledge deficits, especially regarding the IUD, continue to exist for AYA patients. Many participants stated they required EC despite “satisfaction” with their birth control method(s) and most were unaware that the copper IUD could be used as EC. These discrepancies highlight the importance of comprehensive contraceptive education for AYA patients. Enhanced and consistent contraceptive options counseling can help providers ensure that their AYA patients make well-informed decisions about family planning, thus improving their quality of life.

## Plain English summary

In this study, we demonstrate that barriers of access, awareness and knowledge continue to exist for adolescents and young adults (AYA) when it comes to contraception. Specifically, despite awareness about the intrauterine device (IUD), AYA lack adequate knowledge regarding its utility. The results of our study highlight the need for comprehensive contraception educational initiatives. For example, placing an IUD for emergency contraception could then additionally provide ongoing contraceptive benefits. Curricula that highlight the dual use of the IUD could help AYA see the short- and long-term benefits of using the IUD. This study assesses the baseline contraceptive knowledge and attitudes of AYA, which could inform and help healthcare providers tailor the sexual health education they provide their AYA patients. This would ultimately help AYA patients to overcome the barriers they face when choosing contraceptive methods that are best suited for them. This study affirms the current contraceptive knowledge and beliefs of AYA patients and serves as a jumping-off point for education and provision of contraceptive options counseling.

## Introduction/background

The American College of Obstetrics and Gynecology (ACOG) has recommended intrauterine devices (IUDs) as first-line contraceptive choices for parous and nulliparous adolescents [1]. The American Academy of Pediatrics (AAP) endorses the use of IUDs as contraception to parous adolescents and to those who consistently protect themselves against sexually transmitted infections (STI) [2]. IUD use has increased over the past decade; however, overall U.S. IUD use remains low [3–5]. Copper IUDs can also function as emergency contraception (EC), yet its use as such remains limited [6]. Existing research has revealed that young women have limited knowledge about and access to IUDs [7]. Despite its effectiveness, overall use of IUDs in the U.S. remains low. Only 12% of current contraceptive users reported long-acting reversible contraception (LARC) use between 2011 and 2013 [8, 9]. Studies have explored the reasons for the continued low rate of use and insertion of the IUD in adolescents and young adults despite the recognition that the IUD is a safe and effective contraceptive method [10, 11].

Whitaker et al. found that only 40% of 144 female participants aged 14–24 had heard about IUDs; once educated, they began to think positively about IUDs [7]. However, awareness is not enough. In a 2012 study done by Barrett et al., they found that only 39.4% of subjects who had heard about the IUD were able to identify its features [12]. Awareness and perceived knowledge of IUDs among males is low in comparison to condoms and birth control pills [12]. Since male partners can influence the contraceptive decision-making process, it is important that studies are done to understand their perspectives.

This study aims to understand baseline contraceptive knowledge and attitudes of adolescents. This understanding will help healthcare providers improve sexual health education and overcome barriers faced by patients when choosing contraceptives methods that are best suited for them.

## Methods

Subjects were recruited from Staten Island University Hospital’s adolescent clinic. The study was offered to all patients in this clinical setting, which included male and female patients, aged 13 years old to 23 years old. The study was offered to all new and existing patients over a six-month period, from March 2018 to August 2018. Potential participants were provided with a written document containing information regarding the study and provided verbal consent if they chose to participate. They then completed a twenty-minute anonymous survey, written in English, that assessed their knowledge and overall attitudes towards different methods of contraception, with an emphasis on the IUD. Inclusion criteria consisted of age between 13 to 23 years old and the ability to read and comprehend in English.

The survey consisted of five questions regarding sexual history (including sexually transmitted infection history, pregnancy history, contraception use), three questions about emergency contraceptive use, a section on knowledge about birth control methods which consisted of yes/no and true/false/“I don’t know” questions, and a section on knowledge about the copper IUD which consisted of true/false/“I don’t know” questions. The survey also included demographic questions regarding age, gender, educational level, race/ethnicity, and health insurance status.

The primary objective of this study was to determine adolescent and young adult knowledge of the copper intrauterine device (IUD) as a method of both emergency and long-acting contraceptive method. Assuming that the expected prevalence of knowledge of the copper IUD among adolescents aged 13 to 23 years old is 50%, we estimated that a sample size of approximately 100 subjects would provide us with a two-sided 95% confidence interval for the true prevalence that would extend 10% from the observed prevalence. Within this clinical setting, a total of 131 participants completed the survey. Of the completed surveys, 130 completed surveys met criteria for inclusion in this analysis. One subject was excluded because the participant’s age was beyond the study’s range.

The study design received Northwell Health Institutional Review Board approval prior to implementation. Participants provided verbal informed consent prior to completing the survey. Data collection involved investigators entering responses from completed surveys into a password-protected research database (REDCap). Only investigators listed on this study had access to the data.

### Statistical analysis

Demographic and clinical characteristics for the study population were summarized using means with standard deviations for continuous variables and frequencies with percentages for categorical variables. Differences between groups in continuous variables were estimated with independent-sample *t* test. For categorical variables, either Chi-square test or Fisher’s exact test were used as appropriate. All tests were two-tailed and Differences were considered significant at *P* <  0.05. All statistical analyses were performed using SAS software (Statistical Analysis Systems Inc., Cary, NC, USA) Version 9.3.

## Results

There were 99 female participants (76.2%) and 31 male participants (23.8%). The mean age of participants was 18.3 years old. The majority (65.3%) of respondents were aged 18–23 years old and about one third (34.7%) were aged 13–17 years old. A majority of respondents were either in high school (38.5%) or college (44.3%). Demographic results revealed 31.3% Black/African-American, 30.5% Latino/Hispanic, 17.6% White, 3.0% Asian, and 14.5% Other. A majority of respondents had health insurance, either private (25.6%) or public (40.2%).

The majority (80%) of participants were sexually active. The majority (82.8%) reported having partners of the opposite sex, 14.1% reported having with partners of the same sex, and 3.0% reported having both partners of the same and opposite sex. Most (69.5%) participants stated they or their partner were currently using a contraceptive method. Of those using birth control, 71% used condoms, 38% used oral contraception pills (OCP), while only 2.6% used IUDs. Approximately one third (36.4%) of total respondents reported a history of EC use by them or their partner(s). The majority (90.5%) of total respondents reported no history of STIs and 90.4% reported no history of pregnancies in themselves or their partner(s).

Most of the participants surveyed were aware of contraceptive methods. Survey results indicated that 100% were aware of male condoms; 89.9% were aware of female condoms; 92.2% were aware of OCPs; 66.7% were aware of IUDs; 63.3% were aware of hormonal implants; 76.2% were aware of injectable contraceptive hormones; 72.1% were aware of hormonal vaginal rings; and 64.8% were aware of hormonal contraceptive patches. Of those who responded that they had heard of the IUD, 84.9% were females and only 15.1% were males [Table 1]. Of the participants who responded that they had heard of the IUD, 90.7% were sexually active, 72.1% stated that they themselves or their partner(s) were using a form of contraception, and 49.4% stated they or their partner(s) had used EC in the past (*p* <  0.001) (Table 1).

**Table 1:** Demographics by Awareness of IUDs^a^

	Have you heard of the IUD?		***P*** value
	Yes (*n* = 86)	No (*n* = 43)	
**Gender**			
Female	73 (84.9)	25 (58.1)	0.002
Male	13 (15.1)	18 (41.8)	
**Sexual History**			
Sexually Active	78 (90.7)	26 (60.5)	< 0.001
Never Sexually Active	8 (9.3)	17 (39.5)	
**Birth Control Use** (self or partner)			
Yes	62 (72.1)	13 (30.2)	< 0.001
No	24 (27.9)	30 (60.8)	
**EC Use** (self or partner)			
Yes	42 (49.4)	5 (11.9)	
No	43 (50.6)	37 (88.1)	< 0.001

^a^The results reported are frequency counts with percentages in parenthesis

Almost half (49.2%) of participants who responded that they were satisfied with their method of birth control had used EC in the past (*p* <  0.001) (Table 2). Of those with a history of EC use by themselves or their partner(s), 83.0% reported that they or their partner(s) were using a method of birth control (*p* <  0.001) (Table 3). Only 17.8% who reported a history of EC use knew the copper IUD could be used for EC (*p* <  0.001) (Table 3).

**Table 2:** Satisfaction with Birth Control and Emergency Contraception (EC) Use^a^

	Satisfaction with Birth Control			P value
	Yes (*n* = 59)	No (n = 8)	I am not using birth control (*n* = 61)	
**History of EC Use** (self or partner)				
Yes	29 (49.2)	6 (75.0)	11 (18.0)	< 0.001
No	30 (50.8)	2 (25.0)	50 (82.0)	

^a^The results reported are frequency counts with percentages in parenthesis

**Table 3:** History of Emergency Contraception (EC) Use^a^

	History of EC Use (self or partner)		P value
	Yes (*n* = 47)	No (*n* = 82)	
**Birth Control Use** (self or partner)			
Yes	39 (83.0)	36 (43.9)	< 0.001
No	8 (17.0)	46 (56.1)	
**Knowledge that Copper IUD can be used as EC**			
Yes	8 (17.8)	8 (10.0)	< 0.001
No	37 (82.1)	72 (90.0)	

^a^The results reported are frequency counts with percentages in parenthesis

The awareness of the IUD was also specifically assessed by gender, sexual history, birth control use, and EC use. Of those who had heard of the IUD, 90.7% reported history of sexual activity and 49.4% reported history of EC use by them or their partner(s) (*p* <  0.001) [Table 2]. Despite having heard of IUDs, both male and female participants lacked knowledge regarding the utility of the IUD, whether or not they were sexually active. (Table 4) Only 14.1% of those who had heard of the IUD knew that it could be used as EC (*p* <  0.001) (Table 4).

**Table 4:** Awareness vs. Knowledge Assessment of Utility of IUDs^as^

	Have you heard of the intrauterine device (IUD)?		P Value
	Yes (n = 86)	No (*n* = 40)	
**The copper IUD is inserted into the uterus.**			
True	51 (59.3)	6 (15.0)	< 0.001
False	3 (3.5)	0 (0)	
I don’t know.	32 (37.2)	34 (85.0)	
**Insertion of the copper IUD can cause cramping.**			
True	35 (41.2)	3 (7.5)	< 0.001
False	4 (4.7)	1 (2.5)	
I don’t know.	46 (54.1)	36 (90.0)	
**The copper IUD prevents pregnancy when used appropriately.**			
True	62 (72.9)	6 (15.0)	< 0.001
False	2 (2.4)	2 (5.0)	
I don’t know.	21 (24.7)	32 (80.0)	
**The copper IUD does not protect against sexually transmitted diseases.**			
True	52 (61.2)	5 (12.5)	< 0.001
False	5 (5.9)	2 (5.0)	
I don’t know.	28 (32.9)	33 (82.5)	
**Irregular Bleeding is a side effect of the copper IUD**			
True	37 (44.1)	3 (7.5)	< 0.001
False	2 (2.4)	2 (5.0)	
I don’t know.	45 (53.5)	35 (87.5)	
**The copper IUD can be used as a method of EC.**			
True	12 (14.1)	4 (10.0)	< 0.001
False	35 (41.2)	2 (5.0)	
I don’t know.	38 (44.7)	34 (85.0)	
**You can use the copper IUD even if you’ve never had a baby.**			
True	50 (59.5)	3 (7.5)	< 0.001
False	3 (3.6)	1 (2.5)	
I don’t know.	31 (36.9)	36 (90.0)	
**The copper IUD does not require daily reminders.**			
True	52 (61.2)	5 (12.5)	< 0.001
False	4 (4.7)	0 (0)	
I don’t know.	29 (34.1)	35 (87.5)	

^a^The results reported are frequency counts with percentages in parenthesis

Participants were provided with an educational piece at the end of the survey, which stated: “The Intrauterine Device (IUD) is a small T-shaped device about 1 inch long. It is a very effective method of birth control that your health care provider inserts into the uterus. Non-hormonal (copper) and hormonal versions are available. The non-hormonal or copper version can be left in place for up to 10 years. The hormonal version can be left in place for up to 3 to 5 years.” They were subsequently asked if they would use and/or recommend the IUD as a form of birth control. Approximately half of the participants remained neutral despite receiving the education and some provided feedback on their decisions. Some participants listed common misconceptions as their reasons against choosing the IUD in their comment section of the survey. Some participants commented that they still did not have enough knowledge regarding the IUD in general and expressed reluctance to use it or recommend to others.

Participants were also provided with information regarding the copper IUD’s function as form of EC. The statement “Studies have shown that the copper IUD is the most effective form of emergency contraception” was provided to the participants. They were subsequently asked if they would use or recommend the copper IUD as a form of EC. Almost half of the participants remained neutral despite receiving this information and some provided feedback on their decisions. The provided feedback did reveal that some participants did feel like the copper IUD would be a good option for EC after reading the information about the efficacy of the copper IUD.

## Discussion

The results of this study showed that the knowledge base of participants in this study was significantly lacking. When participants were asked about specific IUD contraceptive information, a majority of respondents answered with “I don’t know”. This indicated a gap in the information being presented to this population. Though many claim awareness of the IUD, they failed to understand its function or its side effects. The results of this study were similar to the 2015 study performed by Marshall et al., which found that awareness and perceived knowledge of IUDs among males was low in comparison to condoms and birth control pills [12]. However, the same study had also shown that young men’s perceived knowledge of IUDs was lower than their objective knowledge, whereas this study reveals that most males did not know much about the utility of the IUD [12].

Our results revealing only 2.6% of our participants using IUDs mirrored previous studies that demonstrated the low utilization of IUDs in the United States (3–5, and). All of the study participants who had a history of EC use had used emergency contraception in the oral pill formulation. A significant percentage of participants were unaware that the IUD could also be used as a form of EC. Efficacy should play a role in satisfaction with one’s birth control; however, if EC is being accessed, the birth control method may be clearly ineffective. This was consistent with previous studies that have shown that the use of the copper IUD as EC remained limited [6].

Most participants remained “neutral” after reviewing an education section of the survey on the efficacy of the copper IUD as a contraceptive method. However, the positive responses to the education section of the survey on the efficacy of the copper IUD as a good EC option confirmed the importance of distributing factual written information to adolescents and young adults in order to expand knowledge. Provision of written information should create an opportunity to facilitate this reproductive health decision-making process by stimulating a discussion with their health care provider or health educator.

One strength of this study is that it included male as well as female participants. Another strength of this study is that the survey included questions regarding sexual orientation and gender of sexual partners. These variables have not usually been included in earlier contraception studies.

Our study is not without limitations. One limitation of this study was the small participant size. Our study population was also primarily of one geographical region located in a greater urban community. Further, our survey was only offered in English and required participants to be able to read in English. With a larger and more diverse study population, we might determine other factors involved in the reproductive health decision-making process.

## Conclusion

Barriers continue to exist for adolescents and young adults when it comes to contraception - these include, but are not limited to: access, awareness, and knowledge. The IUD remains the first-line contraceptive method offered as recommended by ACOG and the AAP. This study shows that despite awareness about the IUD, adequate knowledge is lacking among adolescents and young adults regarding its utility. The results of this study highlight the importance of committed and consistent comprehensive contraceptive education interventions for adolescent and young adult patients. Future research should include an assessment of the sources of information used by adolescents and young adults to attain their contraceptive knowledge as well as whether or not they received sexual health education as part of their school curricula. Enhanced contraceptive options counseling can help providers ensure that their patients make well-informed decisions about contraceptive methods, thus improving their quality of life.

## Data Availability

The datasets during and/or analyzed during the current study available from the corresponding author on reasonable request.

## Abbreviations

AAP: American Academy of Pediatrics
ACOG: American College of Obstetrics and Gynecology
AYA: Adolescents and young adults
EC: Emergency contraception
IUD: Intrauterine device
OCP: Oral contraception pill(s)

## References

[1] ACOG Committee Opinion No. 392 (2007). Intrauterine device and adolescents. Obstet Gynecol.

[2] Blythe MJ, Diaz A (2007). Contraception and adolescents. Pediatrics.

[3] Alton TM, Brock GN, Yang D, Wilking DA, Hertweck P, Loveless MB (2012). Retrospective review of intrauterine device in adolescent and young women. J Pediatr Adolesc Gynecol.

[4] Mestad R, Securar G, Allsworth JE, Madden T, Zhao Q, Peipert JF (2011). Acceptance of long-acting reversible contraceptive methods by adolescent participants in the contraceptive CHOICE project. Contraception.

[5] Godfrey EM, Memmel LM, Neustadt A, Shah M, Nicosia A, Moorthie M, Gilliam M (2010). Intrauterine contraception for adolescents aged 14-18 years: a multicenter randomized pilot study of Levonorgestrel-releasing intrauterine system compared to the copper T380A. Contraception.

[6] Seetharaman S, Yen S, Ammerman SD (2016). Improving adolescent knowledge of emergency contraception: challenges and solutions. Open Access J Contracept.

[7] Whitaker AK, Johnson LM, Harwood B, Chiappetta L, Creinin MD, Gold MA (2008). Adolescent and young adult women's knowledge of and attitudes toward the intrauterine device. Contraception..

[8] Daniels K (2015). Current contraceptive use and variation by selected characteristics among women aged 15–44: United States, 2011–2013, National Health Statistics Reports.

[9] Lindberg L, Santelli J, Desai S. Understanding the decline in adolescent fertility in the United States, 2007–2012. J Adolesc Health. 2016; 10.1016/j.jadohealth.2016.06.024. 10.1016/j.jadohealth.2016.06.024 PMC5498007 27595471

[10] Higgins J.A. (2017). Pregnancy ambivalence and long-acting reversible contraceptive (LARC) use among young adult women: a qualitative study. Perspectives on sexual and reproductive health, 49(3):TK. 10.1363/psrh.12025 PMC5597464 28419700

[11] Frost JJ, Lindberg LD, Finer LB (2012). Young adults’ contraceptive knowledge, norms and attitudes: associations with risk of unintended pregnancy. Perspect Sex Reprod Health.

[12] Marshall CJ, Gomez AM (2015). Young men's awareness and knowledge of intrauterine devices in the United States. Contraception..

